# Genomic Relationship Between *Brochothrix campestris* and Its Phages: A Cross-Replicon and Interspecies Perspective

**DOI:** 10.3390/genes16101218

**Published:** 2025-10-15

**Authors:** Sabrina A. Attéré, Laurie C. Piché, Antony T. Vincent

**Affiliations:** 1 Département des Sciences Animales, Faculté des Sciences de L’agriculture et de L’alimentation, Université Laval, Quebec, QC G1V 0A6, Canada; sabrina.attere.1@ulaval.ca (S.A.A.); laurie.piche.1@ulaval.ca (L.C.P.); 2 Institut de Biologie Intégrative et des Systèmes, Université Laval, Quebec, QC G1V 0A6, Canada

**Keywords:** *Brochothrix*, evolution, genomic, genomic island, phage, phage-plasmid

## Abstract

**Background/Objectives**: The bacterium *Brochothrix campestris* is closely related to *Brochothrix thermosphacta*, a known food spoilage agent, and *Listeria monocytogenes*, the causative agent of listeriosis. *B. campestris* garnered attention several years ago because it produces brochocin-C, a bacteriocin capable of inhibiting the growth of certain pathogens. It has been recently suggested that phages play a significant role in the evolution of *B. thermosphacta*, similar to the role they play for *L. monocytogenes*. However, understanding the role of phages in the evolution of *B. campestris* has been challenging because only a draft of genome sequences of a single *B. campestris* strain was previously available. **Methods**: In this study, DNA from the *B. campestris* type strain DSM 4712 was sequenced using Oxford Nanopore and Illumina technologies to obtain complete, high-quality genome sequences. **Results**: The assembly revealed the presence of a plasmid and a phage-plasmid. Additionally, chromosomal analysis identified several genomic islands, including one harboring the brochocin genes, suggesting that these genes may have undergone horizontal transfer. **Conclusions**: This study underscores the potential importance of phages in the evolution of *B. campestris*, and also highlights the need for further research into the interactions between *Brochothrix* species and their associated phages to better understand these complex biological relationships.

## 1. Introduction

The *Brochothrix* bacterium genus comprises two species: *B. thermosphacta* and *B. campestris. B. thermosphacta* strains are known spoilage agents and are frequently found on foods, mainly meats [1]. Currently, few strains of *B. campestris* are known, and they originate from soil or grass [2]. Although *B. campestris* may be considered an environmental bacterium, it is thought to have similar metabolic capacities to *B. thermosphacta*, including the ability to spoil meats [3]. *B. campestris* was studied from the 1990s for its ability to produce a two-peptide bacteriocin, brochocin-C, which has the capacity to inhibit pathogens such as *Listeria* sp. and *Clostridium botulinum* [4,5,6]. Consequently, this species could be of potential biotechnological interest.

Only one genomic sequence of *B. campestris* is currently publicly available (RefSeq: GCF_000525915.1)—the type strain (S3, DSM 4712, ATCC 43754, CIP 102920)—and no publications are associated with it. In addition, the genome of this species was initially sequenced using the IonTorrent technology; therefore, the assembly is fragmented into 128 contigs, making it difficult to study the architecture of the genome.

Recently, Nanopore sequencing of the *B. thermosphacta* type strain (DSM 20171) revealed variability in the chromosome sequences of this species, attributed to prophages (i.e., phage DNA integrated into the bacterial chromosome) and genomic islands [7]. Prophages are known to play an important role in the genomic diversity and adaptation of *L. monocytogenes* [8,9,10], which is the etiological agent of listeriosis and most closely phylogenetically related to the *Brochothrix* genus [11].

Mobile genetic elements are involved in the adaptation and evolution of bacteria [12]. Some of them are extra chromosomal molecules (e.g., plasmids) while others can be integrated into the chromosome (e.g., prophages). In every case, mobile genetic elements may confer multiple selective advantages to their hosts, including virulence and antibiotic resistance [13].

Phage-plasmids were first observed approximately 60 years ago in *Escherichia coli* and were quickly adopted as molecular tools [14,15]. However, they remain relatively obscure because they are less frequently encountered in nature and, consequently, are less well characterized. Briefly, phage-plasmids are temperate phages capable of replicating like plasmids [16]. As such, they possess viral genes responsible for functions such as lysogeny, lysis, virion structure, and DNA packaging. Additionally, like plasmids, they carry functions involved in replication and partitioning. A recent large-scale study revealed that phage-plasmids are present in several bacteria and their size distribution is bimodal, with one peak around 50 kbp and another around 100 kbp [16].

Here, we present the complete genome of the *B. campestris* type strain DSM 4712, sequenced using Nanopore and Illumina technologies. In addition to its chromosome, this strain harbors two circular mobile DNA elements: one plasmid (pDSM4712-1) and one phage-plasmid (ppDSM4712-2). This study underscores how mobile genetic elements, particularly those of phage origin, can shape the *B. campestris* genome.

## 2. Materials and Methods

The type strain *B. campestris* DSM 4712 was obtained from the DSMZ collection (Braunschweig, Germany) and was previously deposited by Talon et al. [2]. The total DNA was extracted with QIAamp PowerFecal Pro DNA Kit (QIAGEN, Toronto, ON, Canada) from a culture grown on Heart Infusion Broth agar medium and incubated for 24 h at 25 °C following the manufacturer’s instructions. The extracted DNA was quantified by fluorescence using the PicoGreen kit (Invitrogen, Waltham, MA, USA) and sequenced on an Illumina NextSeq2000 (2 × 150 bp) and a Nanopore PromethION by Plasmidsaurus (Eugene, OR, USA). The Illumina library was prepared using the SeqWell ExpressPlex 96 library prep kit (SeqWell, Beverly, MA, USA), while the Nanopore library was prepared using v14 library prep chemistry without fragmentation or size selection and sequenced on an R10.4.1 flow cell. Base calling was carried out for Nanopore reads using Dorado version 7.1.4 (https://github.com/nanoporetech/dorado accessed on 25 August 2025) on super-accurate mode. Illumina sequencing reads were filtered with Fastp version 0.23.2 [17] and those from Nanopore were filtered using Filtlong version 0.2.1 by keeping the best 90% of reads above 1000 bp or until only 500 Mbp remained (https://github.com/rrwick/Filtlong accessed on 25 August 2025). A hybrid assembly was performed with Unicycler version 0.5.0 [18]. Default parameters were used for all software unless otherwise specified.

The assembled sequences were initially annotated locally with Bakta version 1.9.1 [19]. Manual annotation with BLASTP against the NCBI nr database was also performed for the coding sequences of pDSM4712-1 and ppDSM4712-2, allowing genes to be categorized according to their possible origins. The protein sequences of ppDSM4712-2 were also functionally annotated using Pharokka version 1.7.5 [20] against the PHROG database [21]. Genomic island prediction was performed with IslandViewer 3 [22]. Maps of pDSM4712-1 and ppDSM4712-2 were constructed with SnapGene software version 7.2 (https://www.snapgene.com). Comparisons between pDSM4712-1 and ppDSM4712-2 and visualization of homologous sequences were performed with EasyFig version 2.2.2 [23]. Copy number of pDSM4712-1 and ppDSM4712-2 was inferred by mapping sequencing reads to the sequences with Bowtie version 2.5.1 [24] and SAMtools version 1.17 [25]. The sequencing depth was calculated with qualimap version 2.3 [26]. The copy number was calculated for each of the replicons by dividing its sequencing depth value by that of the chromosome sequence. CRISPR-Cas systems were detected using CRISPRCasFinder [27]. A BLASTn search was conducted for each spacer against the NCBI nucleotide database (nr/nt).

The complete chromosome sequence of *B. campestris* DSM 4712 has been deposited in DDBJ/ENA/GenBank under the accession number CP175511, and the sequences of pDSM4712-1 and ppDSM4712-2 were deposited under the accession numbers PQ657675 and PQ657676, respectively. Illumina and Nanopore sequencing reads were deposited in the Sequence Read Archive database under accession numbers SRR31926063 and SRR31926062, respectively.

## 3. Results

**Figure 1:**
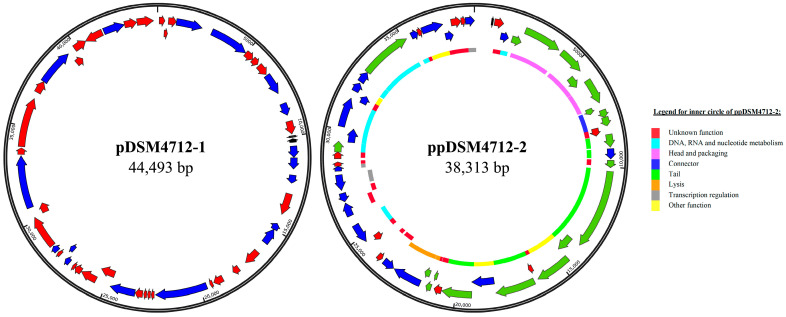
Maps of pDSM4712-1 and ppDSM4712-2. Genes encoding proteins with known functions, which are primarily found on plasmids, are shown in blue. Genes encoding hypothetical proteins are depicted in red, while genes known to be primarily found on phages are highlighted in green. tRNA genes are shown in black. In the inner circle of ppDSM4712-2, each gene is colored according to its associated function.

The aim of the project was to investigate the genome of the *B. campestris* type strain DSM 4712. DNA sequencing and assembly revealed three circular contigs (Table 1): a chromosome and two smaller replicons designated as pDSM4712-1 and ppDSM4712-2 (Figure 1). The adapted abbreviation (pp) before the strain name was chosen for the latter replicon to clearly distinguish phages-plasmids from plasmids, inspired by Pfeifer et al. [16]. By comparing the sequencing depth of the two smaller replicons with that of the chromosome, and assuming that the chromosome is present as a single copy per cell, pDSM4712-1 and ppDSM4712-2 were estimated to be present at 2 to 3 copies per cell.

**Figure 2:**
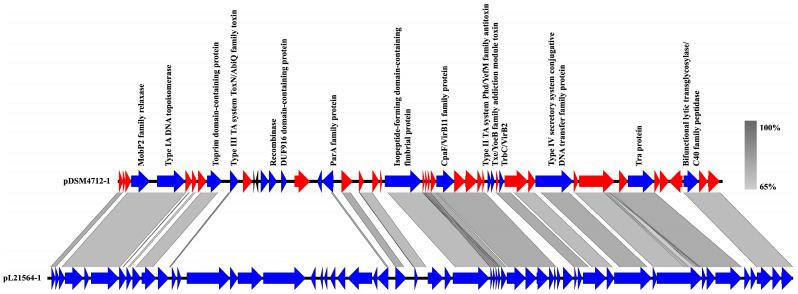
Comparison of the sequences of pDSM4712-1 and pL21564-1. Genes encoding proteins with known functions, which are primarily found on plasmids, are represented in blue. Genes encoding hypothetical proteins are depicted in red. Two tRNA genes are shown in black. Gray shading indicates the homologous regions.

A homology search using the BLASTN tool against the NCBI core_nt database revealed that the best match for pDSM4712-1 is the sequence of plasmid pL21564-1 (Figure 2) from *B. thermosphacta* strain TMW 2.1564, isolated from poultry meat (GenBank: NZ_CP016840). Except for a region that includes a gene encoding a recombinase in pDSM4712-1, both plasmid sequences are collinear, suggesting a common origin or that the plasmids may belong to the same family.

**Figure 3:**
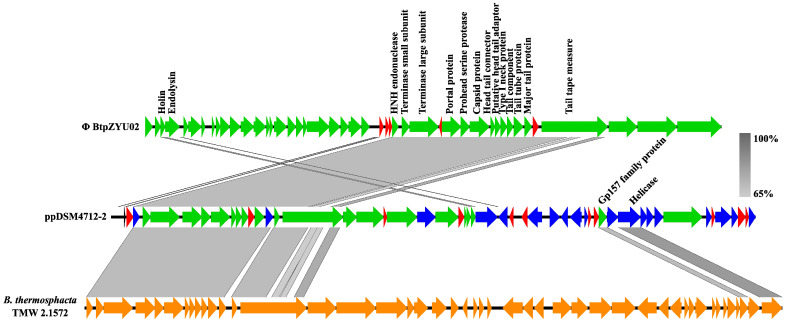
Comparison of the sequences of pDSM4712-1, the phage BtpYZU02, and a region of the chromosome from *B. thermosphacta* strain TMW 2.1572. Genes encoding proteins with known functions, primarily found on plasmids, are shown in blue. Genes encoding hypothetical proteins are depicted in red. Genes known to be primarily found on phages are highlighted in green, while those present on the chromosome are in orange. tRNA gene is shown in black. Only the functions of homologous genes are shown. Gray shading indicates the homologous regions.

Interestingly, the homology search for ppDSM4712-2 revealed a high level of identity with two sequences (Figure 3): that of *B. thermosphacta* phage BtpYZU02 (GenBank: OQ863044.1) and that of *B. thermosphacta* TMW 2.1572 chromosome (GenBank: CP016841.1). Phage BtpYZU02 was isolated from sewage associated with retail pork stalls in China, while *B. thermosphacta* strain TMW 2.1572 was isolated from poultry meat [28]. All three sequences are homologous in a shared region, which primarily contains genes coding for phage structural proteins. Annotation of ppDSM4712-2 revealed that, among the 50 genes encoded by this replicon, 20 and 19 genes have phage and plasmid origins, respectively. However, when searching for homology with TBLASTN, several of the genes were also found in bacterial chromosomes (Table S1); this suggests that ppDSM4712-2 could belong to a prophage family.

Investigation of the presence of genomic islands revealed eight regions with genomic island (GEI) signatures on the chromosome (Figure S1) of *B. campestris* DSM 4712. These GEIs range in size from 4.7 to 56 kbp. They are uniformly distributed along the chromosome, with no preferential integration sites detected. Interestingly, one of these genomic islands (region: 1,951,597..1,977,651) includes genes producing brochocin-C (Table S2). This 26,054 bp region also contains four genes encoding transposases. It is unclear whether these transposases may have been involved in the region’s mobility or whether the region was transferred from another bacterium. A type II-A CRISPR-Cas system with 23 spacers was also discovered in the chromosome (region: 1,273,779..1,281,233). A homology search between the spacer sequences and the CRISPR-Cas++ database revealed no significant results. However, a comparative analysis between *B. campestris* DSM 4712 and *B. thermosphacta* DSM 20171 revealed that both possess a type II-A CRISPR-Cas system with identical direct repeat sequences, but with different spacers (Table S3). BLASTn analysis revealed that none of the spacers in *B. campestris* were linked to known phages. In contrast, spacers in *B. thermosphacta* DSM 20171 matched several phages, notably BtpYZU03 and A9, both of which are known to infect *B. thermosphacta* [29].

## 4. Discussion

Phages are known to play a crucial role not only in regulating bacterial populations but also in shaping bacterial genomes [30]. For instance, phages can integrate as prophages and promote gene mobility through transduction. Genomic sequences of *B. thermosphacta*, a known meat spoilage agent, are becoming increasingly more available, facilitating research to explore the roles and interactions of phages with this bacterium [7,31]. However, the influence of phages on the evolution of *B. campestris*, the only other species in the *Brochothrix* genus, remains largely unexplored.

Using Nanopore sequencing technology, we investigated the genome of the *B. campestris* DSM 4712 type strain and identified three replicons: one large replicon corresponding to the chromosome and two smaller replicons (pDSM4712-1 and ppDSM4712-2). A homology search against publicly available sequences revealed that pDSM4712-1 is homologous to plasmid pL21564-1 found in *B. thermosphacta* TMW 2.1564 [28]. This suggests that these two plasmids may belong to the same family and that members of this family are likely to be found in *Brochothrix* strains. However, the origin of the plasmids and whether they can be transferred between the two species (*B. thermosphacta* and *B. campestris*) remain unclear. Although this element has been highlighted, its activity remains uncertain. Future studies should investigate the potential mobilization capacity of this plasmid.

Characterization of ppDSM4712-2 revealed the presence of several phage-related genes in addition to typical plasmid genes. Consequently, this replicon could realistically be classified as a phage-plasmid hybrid [16]. These hybrid replicons between phages and plasmids have recently been characterized for their ability to mediate gene flow between plasmids and phages [32], as well as to transfer antibiotic resistance genes [33]. To the best of our knowledge, this is the first report of such a replicon in the *Brochothrix* genus. Notably, the region containing the phage genes is homologous to the sequence of phage BtpYZU02, which infects *B. thermosphacta*, as well as to a region of the *B. thermosphacta* TMW 2.1572 chromosome, likely a prophage. This further highlights a potential phage-mediated connection between *B. thermosphacta* and *B. campestris*. It remains unclear whether certain phages capable of infecting *B. thermosphacta* can also infect *B. campestris*, and vice versa. Future studies investigating these phages could provide valuable insights into the evolutionary relationships between these two species. In addition, phage–plasmids may have biotechnological applications, for example, in the development of shuttle vectors, as demonstrated in the bacterium *Leptospira* [34,35]. Similarly, ppDSM4712-2 could be explored as a biomolecular tool in *Brochothrix* species, where no such tools are currently available.

Chromosome analysis revealed that the genes responsible for producing brochocin- C, a bacteriocin with effects against *Listeria* sp., *C. botulinum* and *B. thermosphacta* [4,5,6], are located within a genomic island. This suggests that these genes may have an exogenous origin and were likely acquired through horizontal gene transfer. It has been suggested that the ability of certain bacteriocins to be mobilized may enhance bacterial competitiveness in complex environments, such as the microbiota [36]. Unfortunately, only a few *B. campestris* strains have been isolated to date. Additional strains are needed to determine whether this genomic island is consistently present across all representatives of the species—suggesting acquisition after speciation—or if it is a trait limited to a subset of strains. This investigation is especially relevant given the growing interest in bacteriocins for their antimicrobial effects, particularly in the food industry [37,38,39].

Interestingly, a type II-A CRISPR-Cas system was also identified in the *B. campestris* chromosome. Some strains of *B. thermosphacta* are also known to harbor a type II-A CRISPR-Cas system [31]. However, the number of spacers differs significantly between the system in *B. campestris* (23 spacers) and that in the type strain *B. thermosphacta* DSM 20171 (5 spacers). The search for homology between the spacer sequences and the CRISPR-Cas++ database revealed no significant results, suggesting that additional phages and mobile DNA elements remain to be discovered in *B. campestris*. Although the direct repeat sequences are identical in the two species, none of the spacers are shared. Thus, the presence of different spacers could reflect that the two species inhabit distinct ecological niches. It is currently unclear whether the systems were acquired independently or if they share a common origin. Additionally, the large number of spacers in the *B. campestris* system suggests that it is functional, despite the presence of mobile DNA elements. A study elucidating the functionality and role of the CRISPR-Cas system in *B. campestris* could provide insight into the regulation of mobile DNA elements and phage defense mechanisms in this bacterium.

## 5. Conclusions

Nanopore sequencing of the *B. campestris* DSM 4712 strain allowed us to obtain complete genomic sequences and analyze the replicons present in this genome. The analysis of the two secondary replicons identified a plasmid (pDSM4712-1) and a phage-plasmid (ppDSM4712-2). While pDSM4712-1 has high homology with a plasmid found in *B. thermosphacta*, ppDSM4712-2 is homologous to both a phage infecting *B. thermosphacta* and a chromosomal region, likely a prophage, from another *B. thermosphacta* strain. Although this study revealed a connection between the *B. thermosphacta* and *B. campestris* species, particularly in terms of their mobile genetic elements (plasmids and phages), genomic sequences from additional *B. campestris* strains are needed to further characterize the evolution of the *Brochothrix* genus. At present, only the type strain is available, underscoring the importance of isolating additional *B. campestris* strains in the future. Investigating the potential co-infection of both species by phages would also be of interest, as it could reveal the role of these viruses in promoting gene mobility within this bacterial genus.

## Figures and Tables

**Table 1:** General characteristics of the replicons found in *B. campestris* DSM 4712.

Features	Chromosome	pDSM4712-1	ppDSM4712-2
Length (bp)	2,324,176	44,493	38,313
GC (%)	40.54	35.19	37.85
Number of genes	2145	48	50
tRNA	85	0	1
GenBank	CP175511	PQ657675	PQ657676

## Data Availability

The complete chromosome sequence of *B. campestris* DSM 4712 has been deposited in DDBJ/ENA/GenBank under the accession number CP175511, and the sequences of pDSM4712-1 and ppDSM4712-2 were deposited under the accession numbers PQ657675 and PQ657676, respectively. Illumina and Nanopore sequencing reads were deposited in the Sequence Read Archive database under accession numbers SRR31926063 and SRR31926062, respectively.

## Supplementary Materials

The following supporting information can be downloaded at: https://www.mdpi.com/article/10.3390/genes16101218/s1, Figure S1: Distribution of genomic islands in the *B. campestris* DSM 4712 chromosome; Table S1: Relative frequencies of ppDSM4712-2 coding sequences found in chromosomal, phage, and plasmid sequences; Table S2: List of genes found in the genomic islands of *B. campestris* DSM 4712; Table S3: List of direct repeats (DR) and spacers present in *B. campestris* DSM 4712 and *B. thermosphacta* DSM 20171.

## Abbreviations

The following abbreviations are used in this manuscript:

ATCC	American Type Culture Collection
BLASTN	Basic Local Alignment Search Tool using a Nucleotide query
BLASTP	Basic Local Alignment Search Tool using a Protein query
BP	Base pairs
CIP	Collection of the Institut Pasteur
CRISPR-CAS	Clustered Regularly Interspaced Short Palindromic Repeats–CRISPR-associated protein
DDBJ	DNA DataBank of Japan
DSMZ (DSM)	Deutsche Sammlung von Mikroorganismen und Zellkulturen
ENA	European Nucleotide Archive
GEIs	Genomic islands
Kbp	Kilo-base pairs
Mbp	Mega-base pairs
NCBI	National Center for Biotechnology Information
PP	Phage-plasmid
TBLASTN	Translated Basic Local Alignment Search Tool using a Nucleotide query
TMW	Treated Municipal Wastewater
