# Supplementary material for: Genomic Relationship Between Brochothrix campestris and Its Phages: A Cross-Replicon and Interspecies Perspective
Source: Genes (Basel). 2025 Oct 15;16(10):1218. doi: 10.3390/genes16101218 (PMC12562394; doi:10.3390/genes16101218)
Supplement: Supplementary file 1 [file genes-16-01218-s001.zip › Supplementary_Files/Fig_S1_supplementary data.pdf]

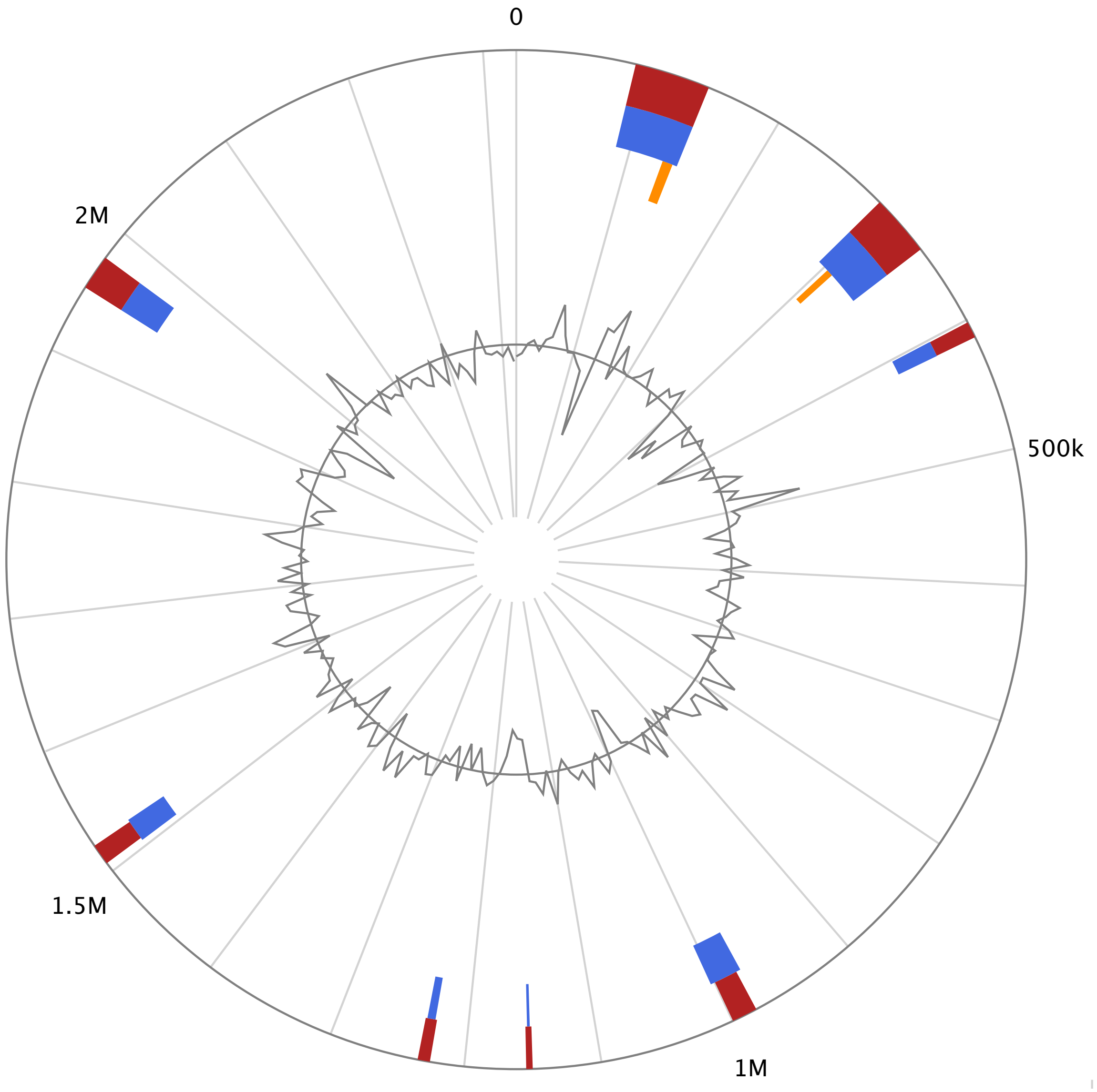

Figure S1. Distribution of genomic islands in the *B. campestris* DSM 4712 chromosome. Red, blue, and orange rectangles represent genomic islands predicted by the "Integrated", "IslandPath-DIMOB," and "SIGI-HMM" methods, respectively. The solid line in the center represents GC content percentage. Chromosomal positions are marked at 0.1 Mb intervals.
